# Warming Drives the Reassembly of Carbon-Sequestering Microbial Communities in Alpine Lakeshore Wetland Without Altering Their Core Metabolic Functional Redundancy

**DOI:** 10.3390/biology15050443

**Published:** 2026-03-09

**Authors:** Zhiyun Zhou, Ni Zhang, Wei Ji, Shijia Zhou, Kelong Chen

**Affiliations:** 1 Qinghai Province Key Laboratory of Physical Geography and Environmental Process, College of Geographical Science, Qinghai Normal University, Xining 810008, China; m13689644891@163.com (Z.Z.); zhangni0224@163.com (N.Z.); jiwei100500@163.com (W.J.); ruye1125@163.com (S.Z.); 2 Key Laboratory of Tibetan Plateau Land Surface Processes and Ecological Conservation (Ministry of Education), Qinghai Normal University, Xining 810008, China; 3 National Positioning Observation and Research Station of Qinghai Lake Wetland Ecosystem in Qinghai, National Forestry and Grassland Administration, Haibei 812300, China; 4 Academy of Plateau Science and Sustainability, People’s Government of Qinghai Province, Beijing Normal University, Xining 810008, China

**Keywords:** Qinghai–Tibet Plateau, simulated warming, *cbbM* gene, carbon-fixing microorganisms, community structure, functional prediction

## Abstract

Climate warming is an important driver influencing soil microbial involvement in carbon cycling. To clarify the responses of carbon-fixing microorganisms in alpine lakeshore wetlands, we conducted a warming experiment using open-top chambers (OTCs) in the Qinghai Lake lakeshore wetland and applied high-throughput sequencing of the *cbbM* gene. The results indicated that warming significantly increased soil temperature and reduced soil moisture, but had no significant effects on soil pH, total carbon, or total nitrogen content. Despite the stability of community α-diversity, warming markedly reshaped the community composition and significantly elevated the relative abundances of dominant taxa including *Ensifer* and *Hydrogenovibrio*. In addition, warming significantly strengthened the assembly process of the *cbbM*-bearing carbon-fixing microbial community, in which heterogeneous selection played a leading role. Redundancy analysis revealed that soil total nitrogen and pH were major drivers influencing the composition of the microbial community. Notably, despite significant fluctuations in taxonomic composition, the functional profile dominated by sulfur oxidation and phototrophy remained unchanged, indicating strong functional redundancy. Overall, *cbbM* carbon-fixing microorganisms in alpine lakeshore wetlands effectively buffered environmental disturbances through functional redundancy and maintained stable carbon metabolic functions, providing scientific evidence for the short-term resilience of carbon sink functions in alpine wetlands under climate warming.

## Simple Summary

Climate warming profoundly affects soil microbial carbon cycling. Using high-throughput *cbbM* gene sequencing, this study explored the responses of carbon-fixing microorganisms to simulated warming in the Qinghai Lake alpine lakeshore wetland. While warming did not significantly alter community α-diversity, it markedly reshaped taxonomic composition, elevating dominant taxa like *Ensifer* and *Hydrogenovibrio*. Warming strengthened deterministic community assembly, primarily driven by heterogeneous selection, with soil pH and total nitrogen as key regulators. Notably, despite significant compositional shifts, the core functional profile remained stable, demonstrating strong functional redundancy. Ultimately, these *cbbM* carbon-fixing microorganisms can effectively buffer short-term thermal disturbances. This study provides critical insights into the short-term ecological resilience and stability of carbon sink functions in alpine wetland ecosystems under future climate change.

## 1. Introduction

Wetlands occupy only 4–6% of the global terrestrial land area, yet they store approximately 12–24% of the organic carbon in terrestrial ecosystems (300–600 Gt), making them a critically important carbon pool [1]. As an important carbon sink on the Qinghai–Tibet Plateau, alpine wetland ecosystems exhibit an acute and heightened sensitivity to climatic fluctuations. With the intensification of global climate warming, rising temperatures are profoundly influencing the assembly of soil microbial communities and their ecological functions [2,3]. Compared to more resilient ecosystems such as established agricultural lands, temperate forests, grasslands, or tundra, soil microorganisms within wetland ecosystems demonstrate a heightened sensitivity to thermal increases, playing a central role in modulating the carbon cycle [4]; in particular, carbon fixation microorganisms (CFMs) carrying the *cbbM* gene directly participate in CO_2_ fixation through photoautotrophic and chemoautotrophic pathways, thereby functioning as a keystone for maintaining carbon balance in wetlands [5].

Temperature serves as a fundamental environmental regulator governing soil microbial growth and metabolic activities and plays a central role in driving critical biogeochemical cycles [6,7]. It has been demonstrated that climate warming exerts significant impacts on soil microbial community composition [8], species diversity [9], and the complexity and stability of microbial interaction networks [10]. Multiple studies have indicated that temperature increases can directly lead to changes in soil microbial community structure [11]. Global-scale meta-analyses further demonstrate that warming generally reduces soil microbial diversity [12], a phenomenon that has been verified in tropical forests [13], temperate forests [14], and grassland ecosystems [9]. In addition, based on warming experiments conducted in the Arctic tundra and alpine meadows of the Qinghai–Tibet Plateau, researchers have observed increases in microbial α-diversity, significant shifts in species composition, and changes in the relative abundances of major groups such as Actinobacteriota and Proteobacteria [3,15]. These structural changes are typically driven jointly by deterministic processes (e.g., environmental filtering) and stochastic processes (e.g., dispersal limitation and ecological drift). Notably, warming can further regulate microbial communities through indirect pathways, such as reducing soil moisture, altering pH, and influencing organic carbon dynamics, vegetation composition, and nutrient availability [4,16]. Changes in soil temperature and moisture conditions caused by global warming often lead to adjustments in plant community structure and ecosystem biomass [17], which subsequently influence the quality and quantity of soil organic matter and feed back to regulate microbial community composition and function [18].

Warming also exerts a profound impact on the functional structure and core metabolic processes of soil microorganisms. Research highlights that climate change not only reconfigures the microbial community structure but also modulates the dominance and distribution of specific functional clusters [19]. For example, long-term warming experiments have found that the expression of key functional genes involved in the carbon cycle (such as those encoding cellulose degradation and carbon fixation) decreases, whereas the abundance of genes related to carbon mineralization increases, indicating a shift in microbial utilization strategies for soil organic matter [20]. However, other studies have demonstrated that warming significantly enhances key metabolic pathways associated with the carbon cycle. By combining long-term field warming experiments with meta-analyses, researchers found that at the gene target level, warming significantly increased the signal intensities of 16.2% of all carbon cycle genes and 19.6% of bacteria-specific carbon cycle genes [21]. Luo et al. analyzed metagenomic datasets from 12 grassland soil communities in the Midwestern United States and likewise reported that warming enhanced several key metabolic pathways associated with the carbon cycle–such as cellulose decomposition and CO_2_ production–as well as denitrification processes within the nitrogen cycle [22]. In addition, genes involved in the phosphorus and sulfur cycles exhibited higher mean expression levels under warming, suggesting a consistent acceleration of nutrient cycling processes [23]. These results imply that the impacts of warming on soil microorganisms are primarily manifested in the restructuring of functional genes and metabolic pathways, rather than solely in shifts in taxonomic composition. Such functional gene reorganization subsequently drives changes in the overall metabolic performance of microbial communities, including alterations in carbon substrate utilization and increases in respiratory entropy [24,25,26]. In addition, warming can indirectly regulate microbial functional expression by altering vegetation types, root exudate composition, and soil available nutrients, thereby forming a more complex plant–soil–microbe feedback mechanism [27,28].

However, whether shifts in community structure inevitably lead to corresponding alterations in ecosystem functions remains a subject of ongoing debate. On the one hand, existing studies support the ‘structure-function synchrony’ hypothesis. For instance, during prolonged warming, changes in microbial community structure are frequently accompanied by marked alterations in soil respiration, enzyme activities, and the abundance of nitrogen-cycling functional genes, suggesting that the response of ecosystem functions is highly synchronous with the turnover of community structure [23,29,30]. These changes may stem from the decline of microbial taxa with lower thermal adaptation or the enrichment of functionally specialized taxa (e.g., thermophiles) [31,32]. On the other hand, increasing evidence suggests that microbial communities may maintain ecosystem function stability through functional redundancy, a phenomenon known as the “decoupling of microbial community structure and function” [33,34,35]. Functional redundancy refers to the capacity of distinct species to perform similar ecological functions, thereby buffering fluctuations in ecosystem processes under environmental disturbances [36]. For instance, under the combined effects of drought and warming, alpine grassland soil microbial communities underwent significant turnover, yet carbon mineralization rates remained stable [37]. Under warming scenarios, factors such as soil desiccation, reduced microbial biomass, and thermal adaptation may offset the expected stimulation, leading to an overall neutral response in soil carbon mineralization [38]. Similarly, in alpine wetlands, carbon-fixing microbial communities may rely on multiple taxa to jointly perform CO_2_ fixation, thereby maintaining the stability of carbon fixation potential during community reassembly. Studies on microbial community assembly mechanisms further reveal that the relative importance of deterministic processes (e.g., environmental filtering) and stochastic processes (e.g., dispersal and drift) determines community structural and functional responses [37,39]. Warming often strengthens deterministic selection, leading to community structural convergence [40]. However, if functional redundancy is high, core metabolic functions may remain unchanged even if significant shifts occur in community composition. Furthermore, the maintenance of functional redundancy may also stem from mechanisms such as physiological plasticity, interspecific interactions (e.g., cross-feeding), and horizontal gene transfer [36,41]. Together, these mechanisms enhance the resilience of microbial communities to environmental change.

The *cbbM* gene encodes the type II RubisCO enzyme and is mainly found in chemoautotrophic and photoautotrophic bacteria, serving as an important biomarker for microbial carbon fixation potential [42]. In alpine wetlands, these microorganisms may be key contributors to carbon sink functions [43]. Despite extensive research on warming impacts regarding organic carbon and nitrogen turnover, there remains a scarcity of data on the assembly mechanisms and stability of *cbbM* carbon-fixing microbial guilds. Given that the lakeshore wetlands of Qinghai Lake represent typical ecotones with high environmental heterogeneity and vulnerability to climate change [44], they were selected as the model system for this study. We employed open-top chambers (OTCs) to simulate warming and integrated *cbbM* amplicon sequencing with FAPROTAX analysis to investigate specific scientific objectives: (1) Does warming induce significant reorganization of the *cbbM*-harboring carbon-fixing microbial community structure? How do its community assembly processes change? (2) Does warming alter their core metabolic functions? (3) Is there a decoupling phenomenon between community structural and functional responses, and what are the potential mechanisms maintaining this pattern? The findings of this study will help elucidate the coupling mechanisms between the structure and function of wetland carbon-fixing microbial communities under climate warming, providing a theoretical basis for carbon cycle modeling and climate adaptation strategies in alpine wetlands.

## 2. Materials and Methods

### 2.1. Site Description

**Figure 1:**
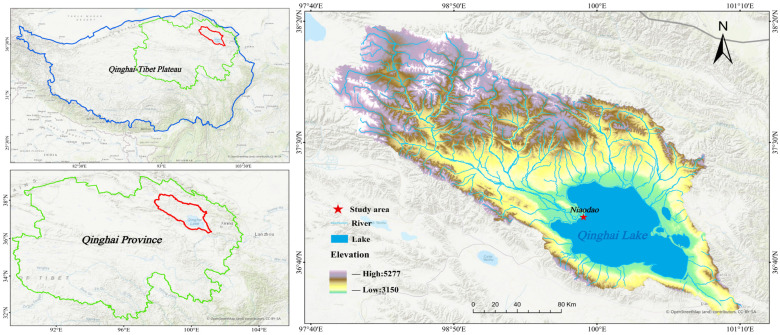
Sampling point distribution. The blue, green, and red lines in the left panels represent the boundaries of the Qinghai-Tibet Plateau, Qinghai Province, and the Qinghai Lake Basin where the Niaodao experimental site is located, respectively.

Field experiments were conducted at the Bird Island Station, part of the Qinghai Lake Wetland Ecosystem National Positioning Observation and Research Station (36°59′ N, 99°57′ E). Situated at the apex of the Buha River delta north of the river mouth, the site follows a northwest-to-southeast gradient with an elevation range of 3194–3226 m (Figure 1). The area is defined by a semi-arid alpine plateau climate, exhibiting distinct continental features such as high aridity, intense solar radiation, frequent gales, and significant diurnal temperature fluctuations. Historical meteorological data indicate a mean annual temperature of −0.7 °C and precipitation of 322.7 mm (occurring primarily from June to September), while annual evaporation reaches 1226.3 mm.

The study area is relatively flat and prone to inundation by lake water. The groundwater is saline, and vegetation coverage is relatively high. The grassland vegetation is dominated by *Leymus secalinus*, *Poa pratensis*, and *Allium przewalskianum*. The soil contains a high proportion of gravel and is classified as sandy loam.

### 2.2. Experimental Design

The warming simulation experimental platform was established at the experimental station in 2018 (Figure S1). Within a 50 m × 50 m area, ten subplots (4 m × 4 m each) were established, utilizing open-top chambers (OTCs) to induce warming. Following three years of continuous warming, soil sampling was conducted in mid-June 2020. The study compared warming treatment plots (NW) against natural control plots (Nck), with five replicate subplots for each group.

Sampling employed a five-point composite method: in each subplot, five soil cores (5 cm diameter, 10 cm depth) were randomly extracted and mixed to form a single composite replicate. This resulted in five composite samples per treatment. All samples were sieved (2 mm mesh) to eliminate roots and debris. Subsamples were immediately frozen in liquid nitrogen for *cbbM* gene sequencing, while the remainder was air-dried for physicochemical analysis.

Considering that the field observation station is located in a remote lakeshore area on an island with extremely limited human access, poor signal reception, and difficulties in power supply, together with the influences of seasonal freeze–thaw subsidence on the plateau and seasonal fluctuations in lake water level, OTCs were installed following the International Tundra Experiment (ITEX) protocol to simulate warming effects (Figure S1).

The passive warming OTCs have advantages such as low cost, simple operation, and high reproducibility, and are widely used to investigate the effects of climate warming on ecosystems, particularly in long-term field observations in alpine regions of the Qinghai–Tibet Plateau. The warming chambers reduce horizontal and vertical air flow to some extent, significantly weakening air turbulence within the chambers. In addition, the fiberglass material has strong transmittance to infrared radiation in solar radiation, thereby increasing the temperature within a specific range inside the chambers. The open-top chambers were constructed from acrylic (polymethyl methacrylate) material with a light transmittance exceeding 92%. The upper surface of the chamber had a diameter of 1.50 m and a side length of 0.87 m, while the lower surface had a diameter of 2.08 m and a side length of 1.22 m. The warming magnitude achieved by the OTCs was approximately 1.3 °C (Figure S1b).

### 2.3. Soil Physicochemical Properties

Soil moisture (Moi) and temperature (Tem) were recorded in situ using a TDR-300 probe (Spectrum Technologies Inc., Plainfield, IL, USA) and an LI-8100 system (LI-COR Inc., Lincoln, NE, USA), respectively. In the laboratory, soil pH was measured in a 1:2.5 soil–water suspension (FE20-FiveEasy pH, Mettler Toledo, Giessen, Germany). Total carbon (TC) and total nitrogen (TN) were quantified using a Vario EL III elemental analyzer (Elemental Analysis System GmbH, Frankfurt, Germany).

### 2.4. DNA Extraction and Polymerase Chain Reaction

Genomic DNA was isolated from soil samples using the OMEGA Soil DNA Extraction Kit (D5625-01, Omega Bio-tek, Norcross, GA, USA). DNA integrity and purity were verified via 0.8% agarose gel electrophoresis and UV spectrophotometry. The *cbbM* gene fragments were targeted using the primer pair 5′-TTCTGGCTGGGBGGHGAYTTYATYAARAAYGACGA-3′ and 5′-CCGTGRCCRGCVCGRTGGTARTG-3′, with PCR protocols following established references. Amplicons were purified (Agarose Gel DNA Recovery Kit) and subjected to Illumina MiSeq high-throughput sequencing at Guangzhou Genedenovo Biotechnology Co., Ltd. (Guangzhou, China).

### 2.5. Statistical Analysis

All statistical computations were executed in R software (v4.1.2). Sequencing depth and alpha diversity were assessed using rarefaction curves from the MicrobiotaProcess package (v4.3.2). Community structural differences were visualized via principal component analysis (PCA) based on Bray–Curtis distances using the vegan package (v2.6.6.1). Biomarkers with significant differences between groups were identified using LEfSe (http://huttenhower.sph.harvard.edu/galaxy/, accessed on 10 July 2025). The FAPROTAX database (Functional Annotation of Prokaryotic Taxa) was used to functionally annotate the OTU table and to analyze the functional groups associated with *cbbM*-carrying microbial communities. We utilized the UpSetR (v1.4.0) and circlize packages (v1.1.0) to generate OTU upset plots and circos plots, respectively. Relationships between environmental factors and microbial communities were examined using Mantel tests (linkET package) (v0.0.7.4) and visualized with qcorrplot (v1.0.12). Redundancy analysis (RDA) was performed using the vegan package (v2.6.6.1). Significant differences were determined by one-way ANOVA (*p* < 0.05). Finally, community assembly processes were inferred by calculating the β-nearest taxon index (βNTI) via the picante package (v1.8.2) and the Raup–Crick metric (RCbray) using the microeco package (v1.11.0).

## 3. Results

### 3.1. Effects of Warming on Soil Environmental Factors in Lakeshore Wetlands

**Figure 2:**
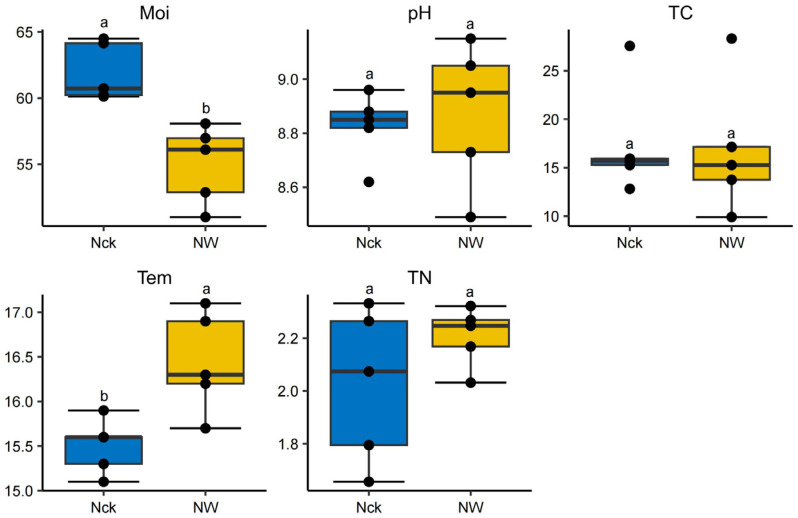
Analysis of differences in environmental factors under different treatments. Distinct lowercase letters denote statistical significance (*p* < 0.05); Moi: soil moisture, Tem: soil temperature, pH: soil pH, TN: total nitrogen, TC: total carbon; Nck: natural control, NW: warming treatment.

As illustrated in Figure 2, experimental warming significantly altered soil physical conditions. Specifically, the NW treatment elevated the mean soil temperature to 16.4 °C (vs. 15.5 °C in Nck; *p* < 0.05) while concurrently reducing soil moisture from 61.9% to 55.0% (*p* < 0.05). Conversely, soil chemical properties, including pH (8.49–9.15), total carbon (9.91–28.32 g/kg), and total nitrogen (1.66–2.33 g/kg), remained statistically stable across treatments (*p* > 0.05).

### 3.2. Effects of Warming on the Community Diversity and Sequence Clustering of cbbM Carbon-Fixing Microorganisms

**Figure 3:**
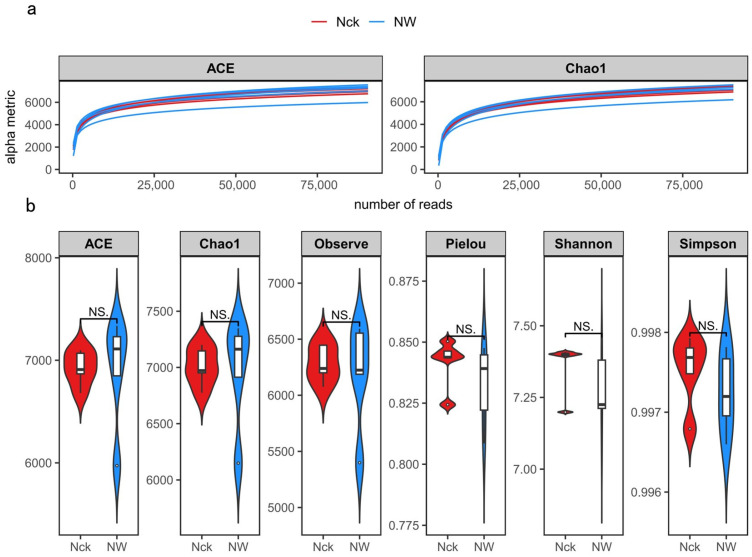
Sample dilution curve (**a**) and alpha diversity index (**b**) of *cbbM* carbon-fixing microorganisms. NS. indicates *p* > 0.05; Species richness indices include the ACE, Chao1, and Observed OTUs indices, while species diversity indices include the Pielou, Shannon, and Simpson indices.

Sequencing adequacy was confirmed by the saturation of rarefaction curves for *cbbM*-carrying organisms (Figure 3a). In terms of alpha diversity (Figure 3b), distinct trends were observed under warming: richness metrics (ACE, Chao1, Observed OTUs) appeared to increase, whereas diversity indices (Shannon, Simpson, Pielou) tended to decline. However, statistical analysis revealed that these shifts were not significant, indicating that warming did not substantially restructure the alpha diversity of the *cbbM* community (*p* > 0.05). 

**Figure 4:**
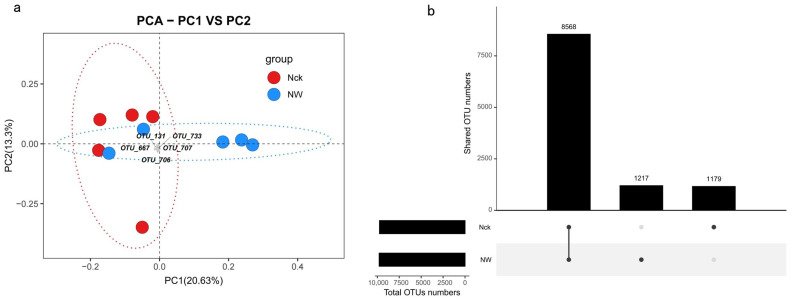
Analysis of carbon-fixing microbial communities; (**a**) principal component analysis of community composition based on Bray–Curtis distances; (**b**) OTU distribution of *cbbM* carbon-fixing microorganisms. Nck: natural control, NW: warming treatment.

Furthermore, based on the PCA (Figure 4a), we observed substantial heterogeneity in the soils of the Qinghai Lake alpine lakeshore wetland. The NW treatment significantly altered the community structure of carbon-fixing microorganisms, causing a deviation from the natural state (Nck). This suggests that key species may reorganize in response to temperature changes, thereby affecting the carbon fixation potential of the ecosystem. Notably, the warming treatment also increased the number of *cbbM*-carrying microbial OTUs to a certain extent. Sequences were clustered into OTUs based on 97% similarity. There were 8568 shared OTUs between the warming treatment and the control group, while the numbers of unique OTUs for Nck and NW were 1179 and 1217, respectively (Figure 4b).

### 3.3. Effects of Warming on the Community Structure of cbbM Carbon-Fixing Microorganisms

**Figure 5:**
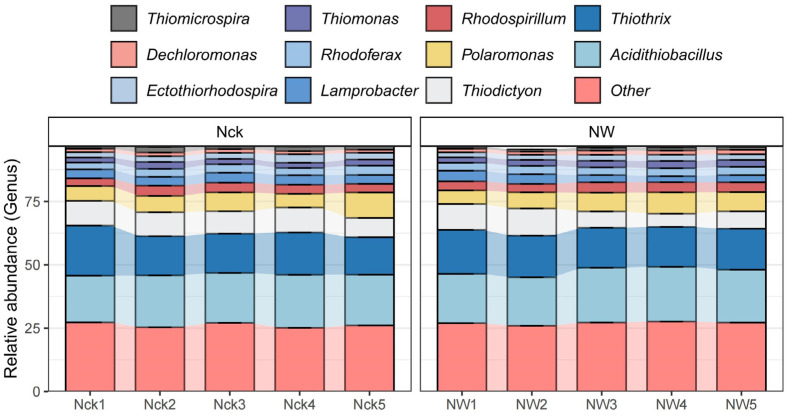
Genus-level relative abundance of major bacterial groups in response to different treatments.

As indicated in Table 1, Proteobacteria emerged as the overwhelmingly predominant phylum within the *cbbM* carbon-fixing microbial community, comprising over 99.9% of the total sequences across all treatments. The combined relative abundance of the remaining phyla did not exceed 1%. While 26.60% of the sequences remained unclassified, we identified 11 genera as dominant taxa (>1%) (Figure 5). Among them, *Acidithiobacillus* had the highest relative abundance (20.20%), followed by *Thiothrix* (16.38%), *Thiodictyon* (8.49%), and *Polaromonas* (7.02%); the combined relative abundance of these four dominant genera exceeded 50%. In addition, *Rhodospirillum*, *Lamprobacter*, *Rhodoferax*, and *Thiomonas* were also important genera, with relative abundances of 3.69%, 3.42%, 3.17%, and 2.41%, respectively (Figure 5).

To investigate the effects of warming on variations in *cbbM*-carrying carbon-fixing microbial communities in lakeshore wetland soils, differential discriminant analysis was performed using the LEfSe method (Figure S2). The results showed that two taxa, *Ensifer* and *Hydrogenovibrio*, were significantly enriched under the warming treatment (*p* < 0.05) (Figure S2). These two taxa belong to different orders, families, and genera, and their relative abundances significantly increased under warming conditions. They served as key indicator species (biomarkers) for distinguishing between treatment groups, providing an important basis for effectively understanding the impact of warming on the structure of *cbbM*-carrying carbon-fixing microbial communities in alpine wetland ecosystems.

### 3.4. Major Functional Groups of cbbM Carbon-Fixing Microorganisms and Their Response to Warming

**Figure 6:**
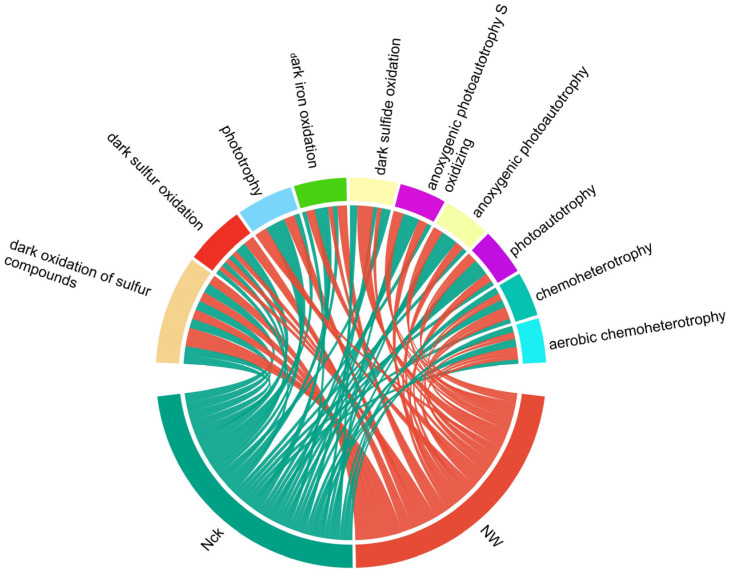
Circos diagram of the relative abundance of different treatment-advantageous functional groups. Nck: natural control, NW: warming treatment.

Functional prediction analysis based on FAPROTAX indicated that the *cbbM* carbon-fixing microbial community in the Qinghai Lake alpine clakeshore wetland involved 44 functional groups. Among them, the top ten functional groups in terms of abundance were: dark oxidation of sulfur compounds (13.99%), dark sulfur oxidation (7.69%), phototrophy (7.24%), dark iron oxidation (6.76%), dark sulfide oxidation (6.11%), anoxygenic photoautotrophy S oxidizing (6.01%), anoxygenic photoautotrophy (6.01%), photoautotrophy (6.01%), chemoheterotrophy (5.72%), and aerobic chemoheterotrophy (5.71%). These top ten core functional groups cumulatively contributed 71.24% to the total functional potential of the community (Figure 6), constituting a metabolic functional profile dominated by sulfur oxidation and phototrophy for *cbbM* carbon-fixing microorganisms.

However, statistical analysis revealed that the functional group composition of these *cbbM* carbon-fixing microorganisms did not show statistically significant differences between experimental treatments (warming vs. control). This result indicates that under the warming conditions established in this study, the core functional structure of the carbon-fixing microbial community in the alpine lakeshore wetland remained relatively stable, and the direct impact of warming on its functional potential was limited.

**Figure 7:**
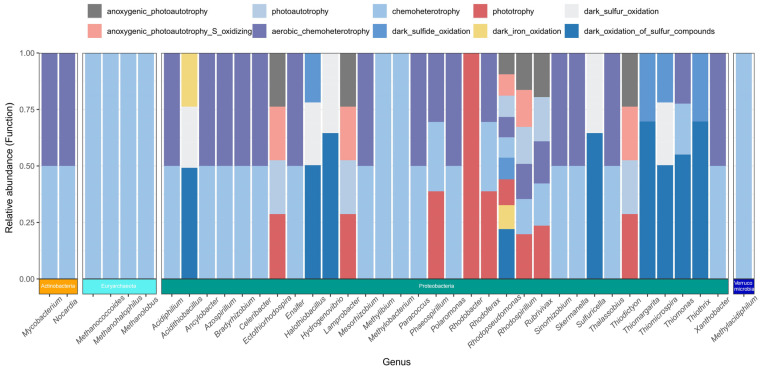
Dominant microbial genera associated with distinct functional groups. The vertical axis represents the relative abundance of specific functional categories, indicated by different colors in the stacked bar chart. The horizontal axis identifies the primary microbial genera responsible for these functions. The colored bars at the bottom indicate the corresponding phyla of these genera.

To further elucidate the microbial carriers of these functional groups, we traced their host classification via sequence back-mapping. The results showed that the microorganisms carrying the aforementioned 44 functional groups were distributed across 39 microbial genera, belonging to 4 different phyla: Proteobacteria, Euryarchaeota, Actinobacteria, and Verrucomicrobia. Among them, Proteobacteria occupied a dominant position in functional carriage and served as the primary host for the vast majority of *cbbM*-related carbon-fixing functions (particularly the highly abundant sulfide oxidation and phototrophic functions). Actinobacteria were specifically involved in chemoheterotrophy and aerobic chemoheterotrophy functions; whereas the functions carried by Euryarchaeota and Verrucomicrobia were more limited, being associated only with chemoheterotrophy (Figure 7).

### 3.5. Relationship Between cbbM Carbon-Fixing Microbial Communities and Environmental Factors

**Figure 8:**
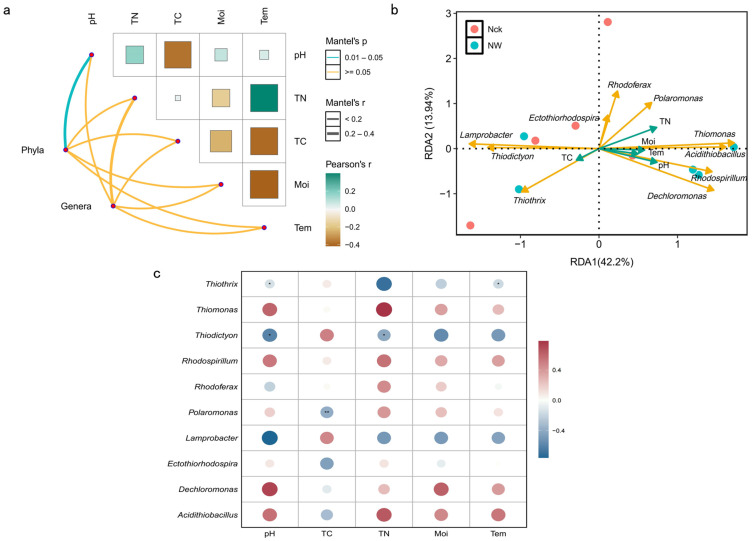
Analysis of the relationships between environmental factors and the *cbbM* carbon-fixing microbial community; (**a**) network analysis illustrating the relationships between environmental variables and the diversity and composition of *cbbM* carbon-fixing microbes; (**b**) redundancy analysis (RDA) identifying the key environmental factors shaping the structure of the *cbbM* microbial community; (**c**) correlation heatmap of *cbbM* carbon-fixing microbial community distribution and environmental factors. where yellow and green arrows represent microbial taxa and environmental variables, respectively; * indicates *p* < 0.05, ** indicates *p* < 0.01.

While warming treatments induced significant shifts in Moi and Tem (Figure 2, *p* < 0.05), other physicochemical parameters (pH, TN, and TC) remained statistically stable. To elucidate the interplay between these environmental variables and the *cbbM* carbon-fixing microbial community, a correlation network heatmap was generated (Figure 8a). The analysis revealed an expected inverse relationship between Moi and Tem, which is consistent with the opposing response trends of the two under warming conditions. Although TC displayed negative associations with Tem and pH, and TN positively correlated with Tem, these trends did not reach statistical significance. Drivers of Community Structure: At the phylum level, soil pH emerged as a significant determinant of *cbbM* community structure, whereas genus-level associations with individual environmental factors were less distinct (Figure 8a). However, RDA identified TN as the primary driver of genus-level community variation, with pH serving as a secondary factor (Figure 8b). Specific genus–environment interactions (Figure 8c) were predominantly negative: *Thiothrix* and *Thiodictyon* were significantly constrained by pH; *Polaromonas* showed a strong negative correlation with TC; and *Thiodictyon* and *Thiothrix* were negatively associated with TN and Tem, respectively (*p* < 0.05).

### 3.6. Effects of Different Warming Treatments on Carbon-Fixing Microbial Community Assembly

**Figure 9:**
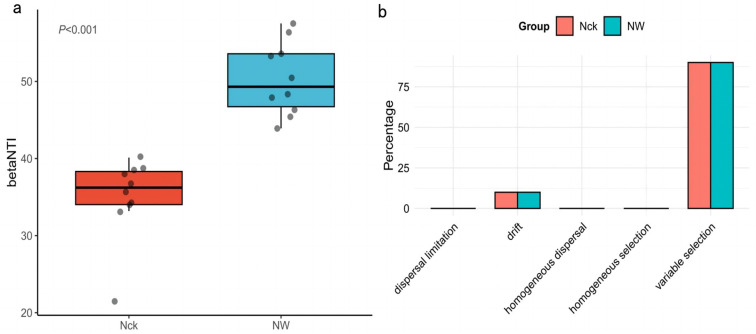
Community assembly of *cbbM* carbon-fixing microorganisms under different treatments: (**a**) β-nearest taxon index (βNTI) indices of different groups; (**b**) distribution of community assembly processes among different groups. Nck: natural control, NW: warming treatment.

The assembly of the *cbbM* community was governed by a combination of deterministic and stochastic processes, with a clear preponderance of deterministic factors (|βNTI| > 2) at the regional scale. Notably, warming significantly intensified these deterministic assembly processes (*p* < 0.001; Figure 9a). Further partitioning of ecological processes based on Bray–Curtis distances (Figure 9b) demonstrated that heterogeneous selection was the dominant force shaping community dynamics across treatments, while ecological drift contributed only minimally. The relative importance of these deterministic and stochastic components remained largely consistent across different warming scenarios (Figure 9b).

## 4. Discussion

### 4.1. Warming Drives Pronounced Changes in Sequence Clustering and Community Architecture of cbbM Carbon-Fixing Microorganisms in Lakeshore Wetlands

Elucidating the mechanisms driving climate-induced shifts in microbial assemblies remains a pivotal theme in global change ecology [45]. In this study, we systematically assessed the response of *cbbM* carbon-fixing communities in alpine lakeshore wetland soils to experimental warming. Our findings reveal that elevated temperatures act as a potent force in reshaping community architecture. OTU distribution results showed (Figure 4b) that there were 8568 shared OTUs between the NW and the Nck treatment, while NW and Nck possessed 1217 and 1179 unique OTUs, respectively, indicating that although warming did not cause a complete turnover of the community, it induced substitution and reorganization of partial taxa. PCA further corroborated this, showing a marked divergence in community structure under the NW treatment (Figure 4a), thereby identifying temperature as a critical determinant of microbial assembly.

Notably, while the overall community structure shifted, the taxonomic hierarchy at the phylum level remained robust. Proteobacteria maintained absolute dominance in both treatments (relative abundance > 99.93%), followed by Rhodophyta, Firmicutes, and Euryarchaeota (Table 1). This result is consistent with multiple studies [46,47,48,49], indicating that Proteobacteria, by virtue of their extensive metabolic diversity, strong environmental adaptability, and efficient resource utilization strategies, continue to occupy a dominant position in wetland ecosystems [50,51]. These findings echo Li et al., who reported Proteobacteria dominance (>93.0%) in degraded alpine meadows on the Qinghai–Tibet Plateau [52]; and Wang et al., who confirmed their pivotal role (34–99%) in carbon fixation across Loess Plateau soils [53]. Most taxa within Proteobacteria prefer low-temperature environments, which may be one of the reasons for their high abundance in alpine wetlands. In addition, microorganisms within this phylum possess diverse metabolic pathways and can participate in the cycling of multiple elements, including carbon, nitrogen, sulfur, and phosphorus, thereby maintaining their ecological advantage in harsh environments [50]. However, unlike the community structure in most wetland ecosystems where Proteobacteria (~40%) and Acidobacteria (~20%) are co-dominant [49,54], the soil microbial community in the Qinghai Lake alpine lakeshore wetlands in this study presented a highly singular composition characteristic, with the relative abundance of Proteobacteria reaching as high as 99.93%, while the proportion of Acidobacteria was extremely low and did not form a dominant group. This discrepancy likely stems from the unique environmental conditions of the Qinghai Lake alpine lakeshore wetland, where high-pH, elevated salinity, and seasonal inundation collectively exert strong environmental filtering pressure that favors the colonization and proliferation of alkali-tolerant, aerobic, or facultative anaerobic taxa within the Proteobacteria. In fact, the main dominant genera in this study, such as *Thiothrix*, *Thiodictyon*, *Polaromonas*, *Rhodospirillum*, and *Lamprobacter* (Figure 5), generally possess the aforementioned physiological characteristics, enabling them to form competitive advantages in harsh environments and ultimately shaping a community pattern absolutely dominated by Proteobacteria.

Unexpectedly, and contrary to initial hypotheses, warming exerted no statistically significant influence on the α-diversity of the *cbbM* guild (Figure 3b). While chronic warming is often linked to substantial shifts in soil microbial diversity, our three-year experiment did not mirror these trends. This observation is supported by Wang et al.’s meta-analysis, which found generally insignificant effects of simulated warming on microbial richness and structure [55]. The absence of variation is likely attributable to the experiment’s relatively brief duration (<5 years). Evidence suggests that microbial diversity is resilient to short-term thermal stress but becomes vulnerable under prolonged exposure (>7 years), particularly in forest and grassland biomes [9,11,56]. As noted by Sun et al., negative impacts on diversity tend to amplify as warming duration extends from 1 to 12 years [12]. Thus, we posit that the limited exposure time in our study induced only minor, statistically undetectable fluctuations. In addition, some studies propose that in regions with moderate temperatures (mean annual temperature ~0–10 °C), microbial species are abundant, niche differentiation is high, and metabolic and enzymatic activities are in a sensitive range, thus responding more significantly to temperature changes; whereas in low-temperature (mean annual temperature < 0 °C) or high-temperature (mean annual temperature > 10 °C) regions, microbial metabolism may be suppressed by the environment and tend toward saturation, thereby weakening the intensity of the response to temperature changes [55,57]. The Qinghai Lake alpine lakeshore wetland in this study has a mean annual temperature of only −0.7 °C, belonging to a typical low-temperature ecosystem, and from a bio-thermodynamic perspective, should exhibit strong sensitivity to warming. Nevertheless, the absence of significant changes in α-diversity under short-term warming suggests that the metabolic activity of the alpine wetland microbial community, under persistent low-temperature constraints, may have approached a state of functional saturation. The overall metabolic flux is insensitive to small temperature increases, leading to the community diversity remaining relatively stable in the short term, while deeper responses may have already occurred at the transcriptional and functional levels, such as gene expression and metabolic flux, which remain to be further analyzed from a multi-omics perspective.

Although overall diversity did not change significantly, warming still increased community richness indices (such as ACE, Chao1) to a certain extent, while reducing evenness indices (such as Pielou, Shannon). Ecologically, this divergence, where the total number of species (richness) increases but their relative abundance distribution (evenness) becomes more skewed, indicates that warming may have promoted the preferential growth of certain specific taxa, forming a “dominant species-led” community pattern (Figure 3b). LEfSe analysis further identified that *Ensifer* and *Hydrogenovibrio* were significantly enriched under warming conditions (Figure S2). These two types of bacteria possess metabolic potential for nitrogen fixation and hydrogen oxidation, respectively. Their enrichment may be related to changes in the soil microenvironment after temperature elevation and the advantages of their metabolic strategies; they have stronger adaptability to high-temperature and drought environments, thereby forming local dominant populations. Although the rapid reproduction of dominant species increased the species richness of the community, their dominance led to a decrease in community evenness. This phenomenon is consistent with predictions from the latest ecological models, emphasizing that environmental stress can strengthen the competitiveness of dominant species through selective filtering, thereby altering the community diversity structure [56,58,59]. Wu et al. also found in a study of the Great Plains grasslands in the United States that under warming conditions, many microorganisms with adaptive traits would survive and outcompete other microorganisms [9]. In summary, although short-term warming did not significantly alter the overall diversity of the carbon-fixing microbial community in the alpine wetland, it has driven community structure reorganization by strengthening the competitive advantage of specific taxa, which may in turn affect the carbon cycling function of the ecosystem.

### 4.2. Warming Did Not Affect the Major Functional Taxa and Core Functions of Soil Microorganisms

Distinct microorganisms can perform similar functions, thereby providing a buffering effect for the entire system. This implies that even if environmental changes alter the community structure, its core functions can remain relatively stable through this buffering mechanism [60]. To investigate whether warming affects the core metabolic processes (e.g., sulfur oxidation and phototrophy) of *cbbM* carbon-fixing microorganisms, and whether changes in community structure are accompanied by synchronous shifts in functional response, this study conducted functional prediction analysis of relevant microbial communities based on FAPROTAX. The results indicated that although warming caused significant reorganization of microbial community structure (Figure 4), there was no statistically significant difference in the core metabolic functional profiles between the treatment and control groups (Figure 6). Specifically, metabolic processes related to sulfur oxidation (e.g., dark oxidation of sulfur compounds, dark sulfur oxidation) and phototrophic processes (e.g., phototrophy, anoxygenic photoautotrophy) dominated the functional composition. Together, they accounted for approximately 53.06% of the predicted functional potential and showed no significant fluctuations under warming conditions. This suggests that although the community structure is sensitive to warming, core metabolic functions did not undergo synchronous changes, exhibiting a distinct “structure-function decoupling” phenomenon.

This decoupling phenomenon likely stems from the high degree of functional redundancy within the *cbbM* carbon-fixing microbial community of the alpine lakeshore wetland. In this study, although warming led to the significant enrichment of certain taxa (e.g., *Ensifer* and *Hydrogenovibrio*) (Figure S2) and caused community turnover at the OTU level (Figure 4b), the microbial carriers performing core metabolic functions were widely distributed. Function-host tracking results showed that key functions such as sulfur oxidation and phototrophy are jointly undertaken by multiple genera, including *Thiothrix*, *Thiodictyon*, and *Rhodospirillum* (Figure 7). These taxa all belong to the Proteobacteria phylum, which is characterized by extremely high metabolic diversity. Therefore, even if warming induces changes in the abundance of some taxa, other taxa possessing similar metabolic potentials can still maintain the original functional flux, thereby buffering the impact of environmental perturbations on overall ecosystem function.

Furthermore, this study found that the community assembly process was dominated by deterministic processes, and warming did not significantly alter the assembly mechanisms (Figure 9). This indicates that environmental filtering plays a continuous role in shaping the community. In this context, functional redundancy may facilitate the maintenance of core functional stability under deterministic filtering pressure [61]. Consistent with findings by Pascual-García et al. and Liu et al. [34,62], this study demonstrates that the *cbbM* carbon-fixing microbial community in alpine wetlands effectively mitigates structural perturbations caused by warming through a “many-to-one” functional carrier pattern (i.e., multiple taxonomic units performing the same function), thereby maintaining the stability of core carbon fixation pathways.

In summary, the results of this study indicate that although short-term warming drove the structural reorganization of the *cbbM* carbon-fixing microbial community in alpine lakeshore wetland, the core metabolic functions did not change significantly due to high functional redundancy within the community; thus, there is a clear asynchrony between structural and functional responses. This finding suggests that when assessing the impact of climate warming on microbe-mediated ecosystem functions, relying solely on structural indices may not be sufficient to fully reflect ecological effects; a comprehensive evaluation combining the functional dimension is essential. Future studies should integrate metagenomics and metatranscriptomics to further verify the maintenance mechanisms of functional redundancy and its stability under long-term warming scenarios at the levels of gene expression and metabolic flux.

### 4.3. Influencing Factors and Interrelationships of cbbM Carbon-Fixing Microbial Communities in Lakeshore Wetlands

The assembly, diversity, and ecological network structure of soil microbial communities are profoundly influenced by their microenvironment and physicochemical properties, with soil temperature, moisture, and pH considered key driving factors [63,64]. Among these numerous factors, pH is widely recognized as the primary driver shaping microbial community composition, particularly bacterial diversity and distribution patterns in wetlands [65,66,67]. Its mechanism of action is mainly reflected in two aspects: Directly, pH influences microbial physiological activities and survival by affecting cell membrane stability, enzyme conformation and activity, and transmembrane proton gradients [68,69]. Indirectly, it affects microbial nutrient acquisition and the expression of metabolic pathway genes by regulating the bioavailability of essential cations and anions in the soil, as well as the solubility and chemical forms of organic matter [65,70].

This study confirms that at the phylum level, the community structure of *cbbM* carbon-fixing microorganisms is significantly regulated by soil pH. A key finding is that pH exhibited a significant negative correlation with two key carbon-fixing functional genera–*Thiothrix* and *Thiodictyon*. Under high pH conditions, the relative abundances of both genera significantly decreased. This phenomenon can be explained from an ecophysiological perspective: as chemo-/photoautotrophic sulfur-oxidizing bacteria, their core metabolic process—the oxidation of reduced sulfides (e.g., H_2_S) coupled with CO_2_ fixation—is highly sensitive to environmental conditions [71]. Soil pH profoundly affects the bioavailability of sulfide by directly regulating its chemical forms. Under acidic to neutral conditions, sulfide mainly exists as electrically neutral hydrogen sulfide (H_2_S) molecules, which have high membrane permeability and are easily absorbed and utilized by microbial cells. However, as the environment becomes more alkaline, the chemical equilibrium shifts toward dissociation, transforming H_2_S into negatively charged hydrosulfide ion (HS^−^) and sulfide ions (S^2−^). These charged ionic forms encounter greater resistance to transmembrane transport, and thus their bioavailability is significantly reduced [72]. Therefore, increasingly alkaline conditions may inhibit the growth and carbon-fixing activity of key sulfur-oxidizing bacteria such as *Thiothrix* and *Thiodictyon* by limiting their access to essential nutritional substrates (H_2_S), ultimately leading to a decrease in their relative abundance in the community and significantly affecting the structure of the *cbbM* carbon-fixing microbial community in lakeshore wetlands.

This finding holds important ecological significance because *Thiothrix* and *Thiodictyon* serve as core bridges connecting the sulfur and carbon cycles. Furthermore, *Thiothrix* can couple sulfur and nitrogen cycles through denitrification, while *Thiodictyon*, as a typical photosynthetic purple sulfur bacterium, includes many species possessing the nitrogen-fixing ability to introduce atmospheric nitrogen into the ecosystem [73,74]. Therefore, by inhibiting these key taxa, pH may trigger a cascade effect: primarily influencing microbial functions related to sulfur and nitrogen transformations, and subsequently reshaping the entire *cbbM* carbon-fixing microbial community structure. This aligns with the findings of Mitsuta et al. using the GJAM model, which identified a “significant positive correlation between nitrogen/sulfur metabolic genes and carbon fixation genes,” collectively suggesting that pH may indirectly influence the carbon-fixing community by regulating the microbial nutrient element interaction network (e.g., C-N-S coupled metabolism) [75].

RDA further revealed that at the genus level, TN is the most important environmental factor affecting the *cbbM* carbon-fixing microbial community. This result resonates with the study by Sui et al. on factors influencing microbial communities in degraded wetland soils in Northeast China, which similarly confirmed that bacterial and fungal community structures are significantly regulated by TN concentration, highlighting the fundamental role of nutrient supply in regulating wetland microbial growth [76]. The analysis showed a significant negative correlation between TN and the relative abundance of the photosynthetic sulfur-oxidizing bacterium *Thiodictyon* (*p* < 0.05). This may reflect that under high-TN conditions, changes in environmental nitrogen forms and supply suppress the competitive advantage of nitrogen-fixing *Thiodictyon*; conversely, other microorganisms capable of directly assimilating exogenous nitrogen with lower metabolic costs exhibit stronger competitive potential. A sufficient external nitrogen supply triggers an energy trade-off in microorganisms, driving a community shift toward microbes employing different nitrogen metabolic strategies. This result suggests that the coupling relationship between nitrogen and sulfur cycles not only regulates the abundance of functional strains but also drives the reshaping of the carbon-fixing microbial community structure at a deeper level; however, the specific molecular metabolic pathways involved remain to be further resolved by multi-omics research.

## 5. Conclusions

The results of this study indicate that although short-term warming drove the restructuring of the *cbbM* carbon-fixing microbial community structure in the alpine lakeshore wetland, it did not significantly alter its core metabolic functions. The stability of the community functional profile is mainly attributed to the high functional redundancy within the microbial community, specifically manifested as key metabolic processes such as sulfur oxidation and phototrophy being jointly undertaken by multiple taxonomic units, thereby buffering the impact of environmental disturbances on ecosystem functions. Meanwhile, although warming enhanced the role of deterministic processes in community assembly to a certain extent, the overall metabolic functions did not shift significantly. Furthermore, soil pH and TN were the most important explanatory factors for the variations in diversity and community structure of the *cbbM* carbon-fixing microbes in the alpine wetland. The results of this study emphasize that in alpine wetland ecosystems, microbial functional redundancy is a key mechanism for maintaining the stability of core carbon fixation functions, and this has important implications for the prediction and evaluation of wetland carbon cycling functions against the background of future climate warming. Nevertheless, given the relatively short three-year duration of the experiment, the observed stability likely reflects short-term ecological resilience in this extreme alpine environment. Long-term monitoring is needed to determine whether prolonged warming will ultimately disrupt this functional stability and induce deeper shifts in community diversity and carbon cycling.

## Figures and Tables

**Table 1:** Phylum-level composition and relative abundance of soil bacteria under various treatments.

Phylum	Nck/%	NW/%
Proteobacteria	99.9302	99.9280
Rhodophyta	0.0256	0.0347
Firmicutes	0.0144	0.0128
Euryarchaeota	0.0188	0.0082
Actinobacteria	0.0027	0.0022
Chloroflexi	0.0007	0.0031
Haptista	0.0007	0.0013
Cyanobacteria	0.0000	0.0007
Verrucomicrobia	0.0004	0.0000
Other	0.0066	0.0091

Nck: natural control, NW: warming treatment.

## Data Availability

The raw data have been uploaded to NCBI, and its BioProject is PRJNA1188821.

## Supplementary Materials

The following supporting information can be downloaded at https://www.mdpi.com/article/10.3390/biology15050443/s1; Figure S1: Overview of the Niaodao study area (**a**) and the configuration of open-top chamber (OTC) warming systems (**b**); Figure S2: Linear discriminant histogram of differential abundance of *cbbM* carbon-fixing microorganisms under different treatments. The horizontal bar chart shows the Linear Discriminant Analysis (LDA).

## Abbreviations

The following abbreviations are used in this manuscript:

OTCs	Open-top chambers
NCK	Natural control treatment
NW	Warming treatment
RDA	Redundancy analysis
βNTI	β-nearest taxon index
PCA	Principal component analysis
TEM	Soil temperature
MOI	Soil moisture
TC	Soil total carbon
TN	Soil total nitrogen
