# Supplementary figures and images for: Warming Drives the Reassembly of Carbon-Sequestering Microbial Communities in Alpine Lakeshore Wetland Without Altering Their Core Metabolic Functional Redundancy
Source: Biology (Basel). 2026 Mar 9;15(5):443. doi: 10.3390/biology15050443 (PMC12984991; doi:10.3390/biology15050443)

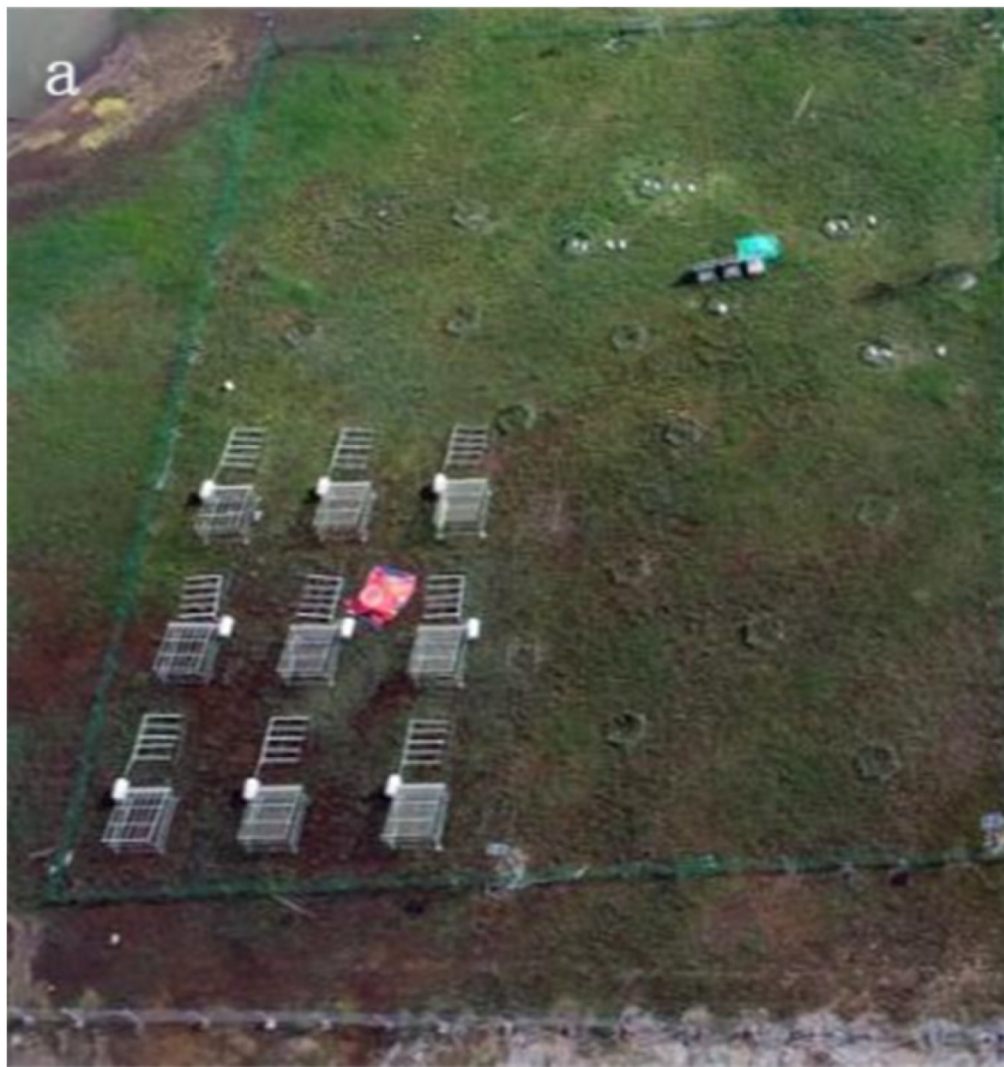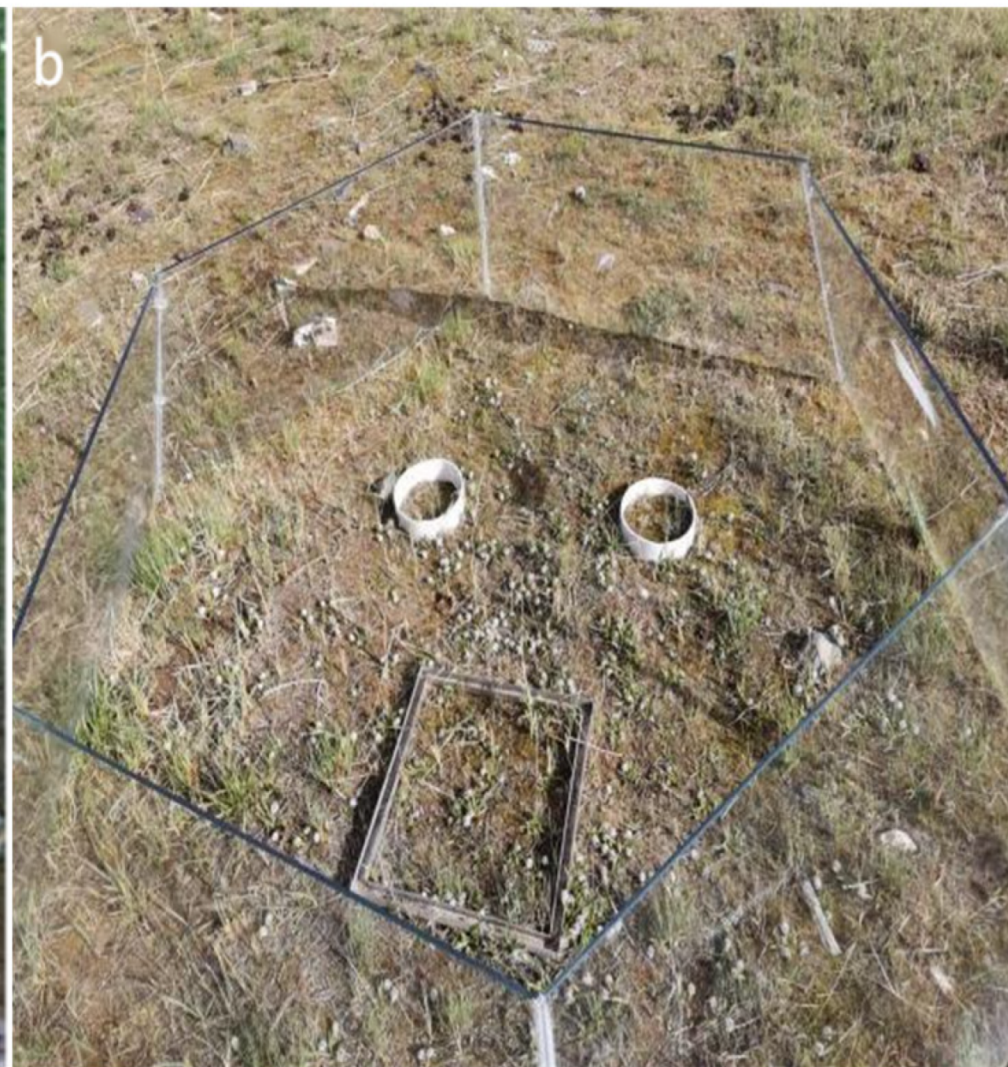

Supplement: Supplementary file 1 [file biology-15-00443-s001.zip › Supplementary material/Figure S1..pdf]

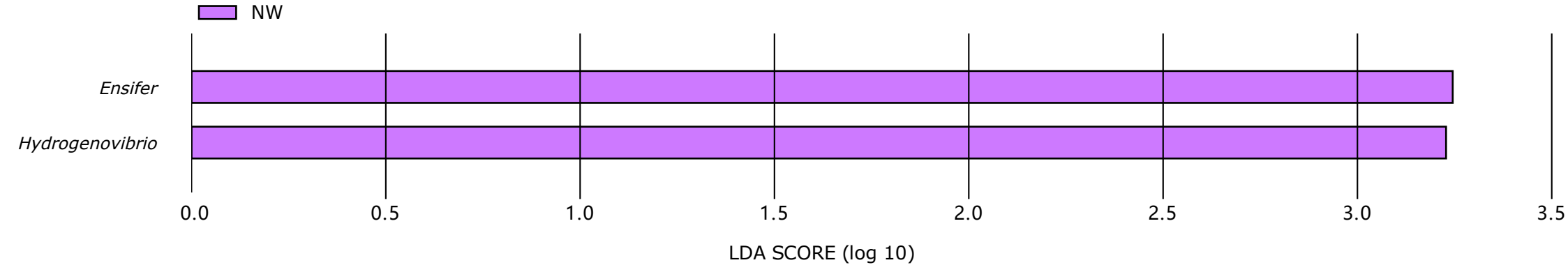

Supplement: Supplementary file 1 [file biology-15-00443-s001.zip › Supplementary material/Figure S2..pdf]
